# The First Ever Reported Case of Primary Synovial Sarcoma of Scalp

**DOI:** 10.1155/2016/5358790

**Published:** 2016-06-29

**Authors:** Biplab Mishra, Saurabh Singhal, Deepak Prakash Bhirud, Nitesh Kumar, Saumyaranjan Mallick

**Affiliations:** ^1^Department of Surgical Disciplines, All India Institute of Medical Sciences, New Delhi 110029, India; ^2^Department of Pathology, All India Institute of Medical Sciences, New Delhi 110029, India

## Abstract

Synovial sarcomas are a rare entity with predilection for extremities and joints. The literature suggests that these tumours are rare in the head and neck region. Very few authors have reported their origin in head. Among the ones occurring in the head region, most of them are found to originate from the parotid area. According to our extensive review of available literature, there has been no reported case of the primary case of synovial sarcoma originating from the scalp convexity. We hereby report one such case in a young female which, by far to the best of our knowledge, is the first ever reported case of a synovial sarcoma occurring on the scalp. The tumour is also the largest ever reported in the literature and posed a great surgical and diagnostic challenge to our team. Through this paper, we recommend that synovial sarcoma should be included as a very rare yet possible differential diagnosis for the scalp tumours.

## 1. Introduction

Soft tissue sarcomas are rare malignancies arising from embryonic mesenchymal cells, thus having varied origin and a wide range of subtypes. They constitute about 1% of all malignancies with an incidence of about 20 per million people and about 8% of cancers in children [1]. Synovial sarcomas are rare subtypes of high grade soft tissue sarcomas and are associated with poorer survival [2]. They have a predilection for extremities and joints and are very rare in head and neck region. There are less than 200 reported cases of head and neck synovial sarcomas in the literature, most of which show predilection for neck and parotid [2, 3]. We present the case of a scalp tumour, which after great diagnostic dilemma proved to be a synovial sarcoma.

## 2. Case History

A 25-year-old woman from a remote village of India presented to the surgery clinic of our hospital with complaint of recurrent swelling on her scalp for the last 6 years. Insidious in onset, painless, and gradually progressive in size, the swelling first occurred on her midscalp and gradually progressed in size over the next 1 year. It was first excised locally by a private practitioner at her hometown. She remained asymptomatic for about two years when she had a recurrence of swelling followed by a second surgical excision at a local hospital. No histopathological diagnoses were made on the excised specimens due to limitation of resources. The patient remained asymptomatic for about a year before she had the second recurrence at the same site but with a more rapid growth and presence of ulceration. She was referred to our tertiary level multispecialty centre for management.

The patient presented to us with a huge swelling rapidly increasing in size. The swelling was large and boggy and was associated with heaviness of the head, pustular discharge, and on and off fever for the last 4 months.

There were no neurological deficits or metastatic symptoms. The patient was married and nulliparous with no obstetric or gynaecologic complaints. There was no significant family history. The patient was depressed due to associated disfigurement and marital problems due to her disease. Viral markers and toxic profile were negative.

**Figure 1:**
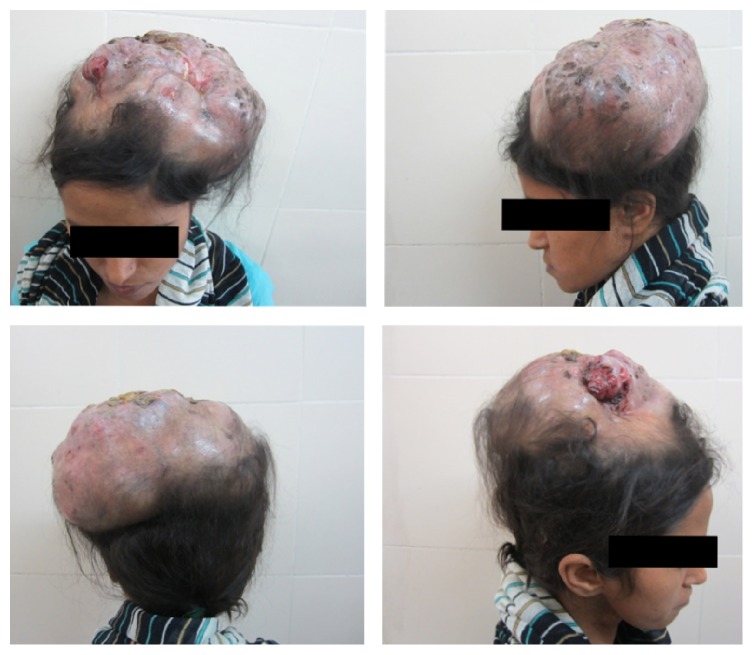
The large bosselated tumour covering almost the whole scalp with multiple ulcers and pus pockets.

Examination revealed a large 25 × 18 cm lobulated, bosselated swelling covering almost the whole scalp with focal ulceration and alopecia. There were multiple pus pockets with foul smelling blood stained pus discharge. Swelling was mildly tender (Figure 1).

There was cervical lymphadenopathy with multiple mobile, nontender, firm lymph nodes in the left submandibular region and posterior triangle. The rest of the general physical and systemic examinations were within normal limits. She had a pulse rate of 89 per minute and blood pressure of 112/78 mmHg. She had a low grade fever (99.2°F).

Multiple cultures were obtained. Pus cultures revealed methicillin sensitive* Staphylococcus aureus* (MSSA). Her blood and urine cultures and further wound swab cultures came sterile. Her fever responded to antibiotics.

Multiple trucut biopsies taken from the scalp swelling came inconclusive. Incisional biopsy was thus taken which showed fibrocollagenous tissue with chronic inflammatory infiltrate and extensive areas of necrosis. There was no evidence of malignancy in any of the histopathological reports. Excisional biopsy of posterior cervical lymph nodes suggested reactive changes.

**Figure 2:**
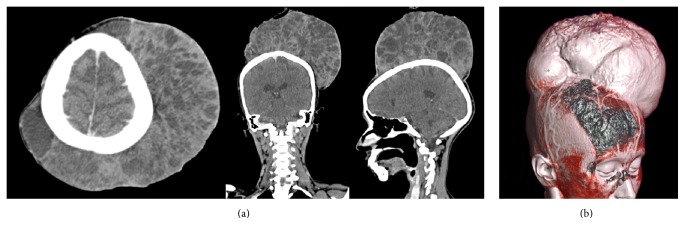
Computed tomography (CT) scan with 3D reconstruction showing highly vascular tumour with clean surgical plane with underlying calvarium.

The patient underwent contrast-enhanced computed tomography (CECT) of head and neck with 3-dimensional reconstruction (Figures 2(a) and 2(b)). Reports suggested a large and highly vascular heterogeneous tumour on scalp with necrotic and cystic areas. There were clear surgical planes with underlying cranial bones with no signs of invasion.

With the provisional diagnosis of recurrent scalp tumour with probable malignant or inflammatory pathology, excision under general anaesthesia was planned. Challenges were to achieve negative surgical margins in a highly vascular and huge scalp tumour and to cover the large scalp defect thus created.

**Figure 3:**
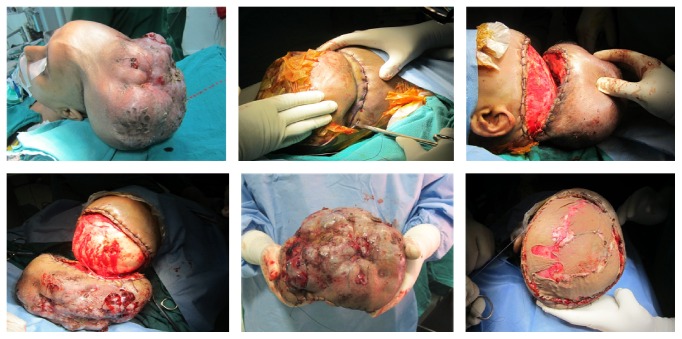
Surgical excision of tumour and coverage of scalp with split thickness skin graft. Note that, with the use of hemostatic sutures, blood loss could be minimised.

With the patient in supine position, the tumour was well painted and segregated with sterile drapes. The head was placed flexed for adequate exposure of the tumour tissue. With high vascularity of the tumour, it was dissected off the cranium with haemostatic sutures applied all around on the tumour edge and with a 2 cm margin on the normal scalp tissue. The raw area thus created was covered with split thickness skin graft harvested from the left thigh. The procedure was uneventful with less than 300 mL of blood loss (Figure 3).

**Figure 4:**
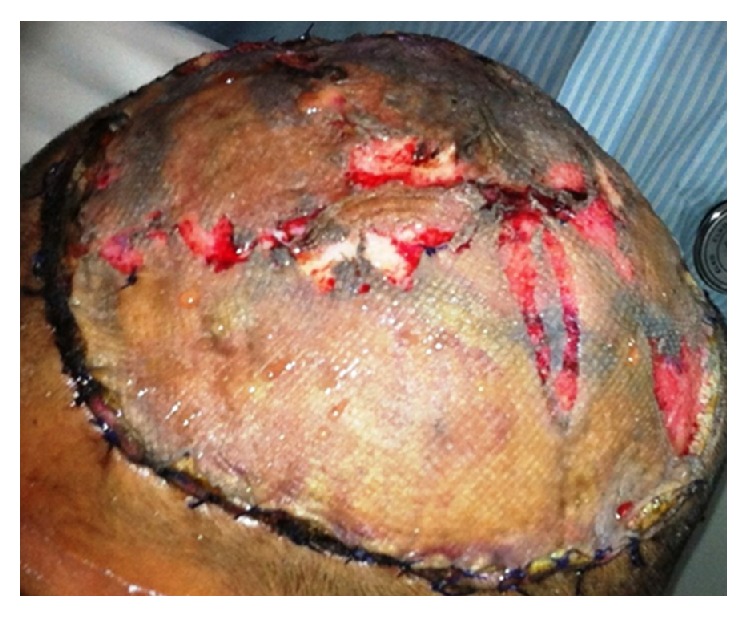
Two-week follow-up revealed healthy graft with granulation tissue and >90% uptake.

The patient made uneventful recovery and was discharged on postoperative day seven with almost 90% graft uptake. Two-week follow-up revealed healthy graft tissue (Figure 4).

**Figure 5:**
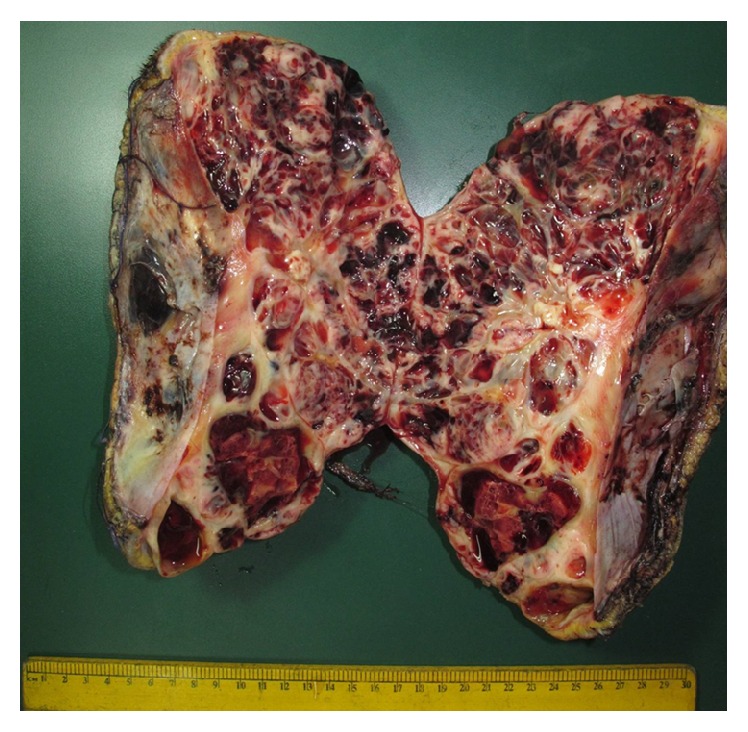
Histopathological specimen of excised tissue revealing the large size of the tumour.

**Figure 6:**
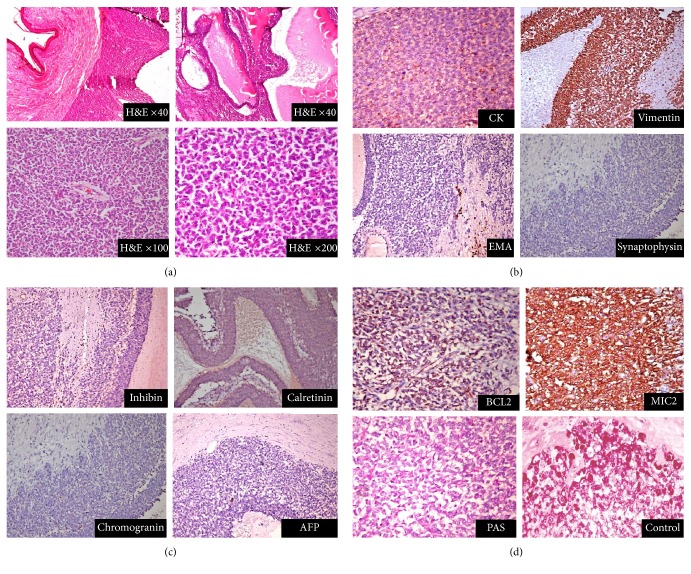
H&E staining revealed poor differentiation and the tumour was immunopositive for vimentin, cytokeratin (focal), Bcl2, and MIC2.

Histopathology of the excised specimen revealed a solid cystic tumour with areas of necrosis. The size of the tumour in excised specimen was 24.5 cm × 12 cm × 10.5 cm (Figure 5). The tumour consisted of round to oval cells, moderate pleomorphism, and increased mitosis. Tumour cells were immunopositive for vimentin, cytokeratin (focal), Bcl2, and MIC2 while they were negative for chromogranin, synaptophysin, inhibin, calretinin, and alpha-fetoprotein (Figures 6(a)–6(d)). The above findings were suggestive of poorly differentiated synovial sarcoma. Positivity for t(X;18) translocation was seen in about 30% of cells and thus supported the diagnosis. All surgical margins were free of tumour.

**Figure 7:**
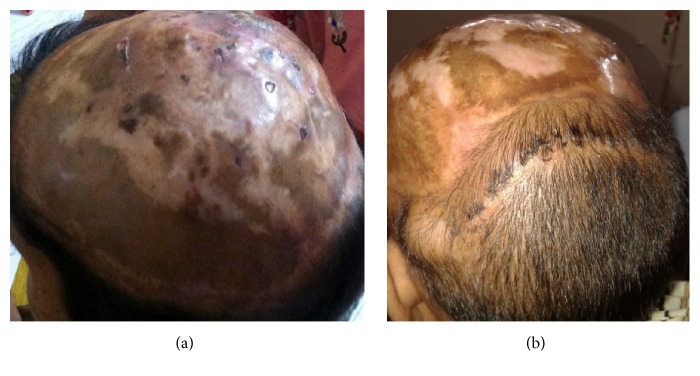
Follow-up showing completely covered scalp tissue (a) and use of tissue expander on the peripheral scalp (containing hair follicles) to cover the bare area (b).

She received postoperative locoregional radiotherapy after complete take of skin graft and epithelisation of all raw areas. A repeat PET-CT at 24 months showed no focus of recurrence of disease. On a 29-month follow-up after surgery, the patient was asymptomatic with no recurrence (Figure 7). As the graft tissue contraction occurred, she was given temporary cosmetic coverage of the scalp with artificial wigs. Further, tissue expanders are being used to expand the remaining peripheral normal scalp tissue which will be used to cover the bald area (treatment with tissue expander is shown in Figure 7). She is also planned for trial of hair transplantation on the remaining grafted skin lacking hair follicles.

## 3. Discussion

Sarcomas of head and neck origin account for <10% of all sarcomas and about 1% of all primary head and neck malignancies [2, 4, 5]. Soft tissue sarcomas constitute 80% of all sarcomas [2]. Synovial sarcomas in turn consist of less than 10% of all soft tissue sarcomas [2]. The peak incidence of synovial sarcoma is in the third decade, common presentation being a young adult presenting with sarcomatous tumour of an extremity [1]. About one-third of synovial sarcomas occur under twenty years of age; however, they still are the second-commonest sarcomas after rhabdomyosarcoma in adolescents and young adults [1].

Synovial sarcoma is a relatively rare subtype of soft tissue sarcoma with histologic resemblance to synovial cells, thus the name. The morphologic subtypes are divided into monophasic and biphasic according to the presence or absence of differentiation into glandular epithelium and a third poorly differentiated subtype. The most characteristic and hallmark genetic alteration is the chromosomal translocation t(X;18)(p11.2;q11.2) [6]. The SYT gene (Chr 18) fuses with SSX1 (Chr X) to form biphasic tumour while fusion with SSX2 leads to monophasic tumour. The latter is associated with longer metastasis-free survival [7, 8]. Stage, tumour size, tumour location, and age at diagnosis are the major factors predicting cancer-specific survival [9, 10]. Despite pathological grading into differentiated and poorly differentiated varieties, they have propensity to locally invade and metastasize and should always be considered and managed as high grade sarcomas [9, 11].

Synovial sarcoma rarely presents on trunk and regions of head and neck. The latter contributes 2.5–3.7% of all synovial sarcoma cases [3, 12, 13]. The first such case was reported by Jernstrom in 1954 [14]. Majority of such synovial sarcomas tend to occur in neck region. In a major series of 36 cases along with analysis of another 28 cases reported in the English literature [3], only 1.9% of all synovial sarcomas occurred in head region and mainly involved temporal and parotid regions, cheek, infratemporal fossa, and mastoid areas. There were rare cases involving other areas of head and none was found to involve convexity of scalp [3, 12, 13, 15, 16].

Based on extensive literature search, our case is the largest case of synovial sarcoma of head and neck ever excised [3, 12, 13, 15]. Before this, the largest tumour ever excised was from cheek and was 18.5 cm × 15 cm × 13.5 cm [3, 17] whereas our tumour was much larger as shown in Figure 5.

Surgery is the mainstay of treatment [18]. However, achieving negative surgical margins in regions of head and neck is very difficult and poses a great challenge to the surgeon. This was a major challenge to our team as well. This results in higher recurrence rates and worse prognosis in head and neck sarcomas [19]. Covering the defect created after excision of large tumours is another challenge. Calvarium has a rich vascular supply and is a potential candidate for split thickness skin graft. Rotational or free flaps are other possible options.

The role of adjuvant therapies is debatable. Chemotherapy takes care of the microscopic cancer cells locally and those disseminated systemically. Locoregional radiotherapy is important to reduce the chances of local recurrence and also takes care of the microscopic or sometimes macroscopic positive surgical margins.

The outcome of a well differentiated tumour with adequate surgical excision and aggressive adjuvant therapies is fairly good with survival rates in the range of 60% [2, 16]. Recurrence is fairly common and close follow-up is required for early detection of recurrence and metastasis, if any.

## 4. Conclusion

As per our extensive search of the literature and the unique presentation of our patient, we can affirmatively say that this is, in most likelihood, the first ever reported case of synovial sarcoma of scalp origin and indeed the largest primary synovial sarcoma of head and neck region ever excised. We emphasise the need of including synovial sarcomas as one of the rare differential diagnoses of head and neck sarcomas.

## Abbreviations

POD: Postoperative day.

## Additional Points

The key message of this paper is to report that this is the first ever reported case of a synovial sarcoma of scalp origin. The authors, thus, recommend the inclusion of synovial sarcoma as one of the rare differential diagnoses for scalp tumours.
